# Stakeholder perspectives on scaling up medical device reprocessing: A qualitative study

**DOI:** 10.1371/journal.pone.0279808

**Published:** 2022-12-30

**Authors:** Rachel Hennein, Emily Goddard, Jodi D. Sherman

**Affiliations:** 1 Department of Epidemiology of Microbial Diseases, Yale School of Public Health, New Haven, CT, United States of America; 2 Yale School of Medicine, New Haven, CT, United States of America; 3 Department of Social and Behavioral Sciences, Yale School of Public Health, New Haven, CT, United States of America; 4 Department of Anesthesiology, Yale School of Medicine, New Haven, CT, United States of America; 5 Department of Environmental Health Sciences, Yale School of Public Health, New Haven, CT, United States of America; University of Miami, UNITED STATES

## Abstract

**Background:**

The United States health care sector is one of the largest polluting industries, which has significant adverse effects on human health. Medical device reprocessing (MDR) is a sustainability solution that has the potential to decrease hospital waste, cut carbon emissions, reduce spending, and improve supply chain resiliency; however, only a small proportion of FDA-approved devices are actually reprocessed. Thus, we conducted a qualitative study to understand barriers and facilitators of scaling up MDR.

**Methods and findings:**

We conducted in-depth interviews with 17 stakeholders (exceeding thematic saturation) at a large academic health system in New England and national MDR organizations. We also collected observations through site visits at the health system. We recruited participants from June 2021 to April 2022 through purposive sampling. Using an analytic approach guided by the Consolidated Framework for Implementation Research, we applied inductive and deductive codes related to key implementation constructs. We then conducted a thematic analysis and identified five overarching themes related to barriers and facilitators of MDR. First, respondents explained that regulatory bodies and original equipment manufacturers determine which devices can be reprocessed. For example, some respondents described that original equipment manufacturers use tactics of forced obsolescence that prevent their devices from being reprocessed. Second, respondents explained that MDR has variable compatibility with hospital priorities; for example, the potential cost savings of MDR is compatible with their priorities, while the perception of decreased functionality of reprocessed medical devices is incompatible. Third, respondents described that physician preferences influence which reprocessed devices get ordered. Fourth, respondents explained that variable staff knowledge and beliefs about MDR influence their motivations to select and collect reprocessable devices. Lastly, respondents emphasized that there was a lack of infrastructure for evaluating and maintaining MDR programs within their health system.

**Conclusions:**

Based on our findings, we have outlined a number of recommendations that target these barriers and facilitators so that the environmental and financial benefits of MDR can be realized at this health system and nationally. For example, implementing federal policies that prevent original equipment manufacturers from using tactics of forced obsolescence can facilitate the scale-up of MDR nationally. Additionally, providing life cycle assessments that compare the environmental effects of single-use disposable, reprocessable disposable, and reusable devices could facilitate health systems’ purchasing decisions. Creating and disseminating audit and feedback reports to hospital staff might also facilitate their continued engagement in the program. Lastly, hiring a full-time program manager that leads MDR programs within health systems could improve program sustainability.

## 1. Introduction

The United States (US) health care sector is one of the largest polluting industries, responsible for 8.5% of national greenhouse gas (GHG) emissions and similar fractions of toxic air emissions [1]. Health care emissions impact human health indirectly through pollution-related disease and climate change-related death and disability [1, 2]. In 2018 alone, US health care emissions resulted in a loss of almost 400,000 disability-adjusted life years [1]. Efforts to mitigate the adverse impacts of health care pollution must include the supply chain, which contributes about 80% of total US health sector GHG emissions on a life cycle basis from natural resource extraction, manufacturing, packaging, transportation, and eventual waste disposal management [1–5].

Medical device reprocessing (MDR) is a sustainability strategy targeting the supply chain to decrease hospital waste, costs, and emissions, and improve resilience. MDR is a third-party industry that collects single-use medical devices, then disassembles, decontaminates, refurbishes and reassembles, tests for functionality, repackages, and re-sterilizes, so that they can be safely resold and reused [6]. These procedures require approval by the US Food and Drug Administration (FDA). MDR extends the lifetimes of disposable products, allowing for a limited number of reuses that are carefully tracked, and vary depending on the device. MDR can facilitate cost savings for the health system by selling single-use devices back to the vendor as well as purchasing reprocessed devices, and it can reduce pollution from de novo natural resource extraction, manufacturing, and waste disposal. In 2018, it was estimated that MDR in the US, Canada, and Europe reduced hospital solid waste by almost 7,100 tons and generated cost savings of more than $470 million, though GHG savings remain unknown [7]. MDR can also improve the resiliency of supply chains, as experienced by programs that reprocessed personal protective equipment during the SARS-CoV-2 pandemic due to global supply chain shortages [8]. Given these benefits of MDR, the market size of this industry is projected to grow from $1495 million in 2022 to $3253 million in 2028 [9].

Despite these advantages, the uptake of MDR in health systems globally has been limited. While the exact figure is unknown, of all medical devices manufactured, approximately 2% are estimated to be reprocessible, and only a small fraction of those eligible devices are recovered [6, 7]. Scaling up MDR requires an understanding of barriers and facilitators to uptake [7]. Thus, we used qualitative research methods to investigate the acceptability and feasibility of MDR from the perspectives of stakeholders affiliated with an academic medical center in New England and national MDR organizations.

## 2. Methods

### 2.1 Conceptual framework

The Consolidated Framework for Implementation Research (CFIR) is widely used for characterizing constructs that influence the implementation of quality improvement initiatives [10, 11], such as MDR. CFIR includes five domains: 1) intervention characteristics, 2) outer setting, 3) inner setting, 4) individual characteristics, and 5) process [10]. With respect to the present study, outer setting refers to characteristics outside of the health system that could influence implementation of MDR, such as FDA regulations and medical device design by original equipment manufacturers (OEMs). The inner setting includes aspects of the health system, such as infrastructure for MDR. Individual characteristics refers to hospital stakeholders’ knowledge, beliefs, and motivations for engaging in MDR. Intervention characteristics are aspects of MDR that influence how it is adopted. Lastly, implementation process refers to how MDR is implemented in the hospital, such as strategies to engage staff.

### 2.2 Setting and participants

This study took place at Yale New Haven Health System, a large academic non-profit health care entity located throughout Connecticut and parts of New York and Rhode Island. Affiliated institutions provide a range of services, from quaternary to primary care, to diverse patient populations. This health system employs about 26,000 people, and is affiliated with Yale School of Medicine. Yale New Haven Health System has contracts in place to reprocess certain medical devices, such as tourniquet cuffs and laparoscopic electrocautery instruments, but does not currently have a system-wide MDR program across all eligible devices. This study focuses on MDR for single-use devices that are used in operating rooms at Yale New Haven Health System, as operating rooms constitute more than 40% of hospital expenses and 30% of hospital waste disposal volume [12]. Thus, situating our study within operating rooms at this health system provides the opportunity to learn from their current MDR initiatives, including barriers and facilitators to scaling up these programs across all clinical areas within the health system.

Study participants included stakeholders at the health system and national MDR organizations. We used purposive sampling to ensure representation of ideas from various stakeholders [13]. We included clinical stakeholders to learn about how reprocessed medical devices are used and disposed of in operating rooms. We also included administrative stakeholders from Corporate Supply Chain, Contracts, Materials Management, and Central Sterile departments because they play a role in deciding which medical devices get reprocessed for the health system. We included industry stakeholders from a third-party MDR vendor and the Association of Medical Device Reprocessors (the global trade association of the MDR industry) to better understand their perspectives on working with health systems and the FDA to set up and maintain MDR programs. We continued sampling until thematic saturation was achieved [14].

The Institutional Review Board at Yale University approved our study (ID: 2000030501). All participants provided verbal consent. We reported all findings using the Consolidated Criteria for Reporting Qualitative Research [15].

### 2.3 Data collection

We conducted multiple site visits to explore the context of MDR in the hospital. RH (a doctoral student) and JS (an anesthesiologist with 14 years of experience in this health system) walked the route of reprocessed medical devices and had informal conversations with hospital staff in order to identify key process steps and relevant stakeholders. We recorded these observations through detailed field notes.

We conducted in-depth interviews using a semi-structured interview guide that was designed by two authors (RH and JS). We included questions from each of the five CFIR domains [16]. After obtaining informed consent, a doctoral student (RH) conducted and audio-recorded interviews on an online video-conferencing platform. RH took detailed notes after each interview to describe factors not captured by audio recording (e.g., participant’s setting). RH wrote reflections after each interview, and met regularly with JS to discuss emerging themes, identify topics for discussion in subsequent interviews, and monitor thematic saturation [17]. All interviews were transcribed verbatim and reviewed for accuracy.

### 2.4 Data analysis

We uploaded all transcripts and field notes into NVivo (QSR International). Our coders included a doctoral student (RH) and a masters student (EG) with prior qualitative research experience. First, we coded three transcripts according to CFIR construct definitions and added inductive codes that were not captured by the CFIR. We then met to discuss discrepancies between codes until consensus was reached, revised the codebook, and coded the remaining transcripts accordingly. The codebook is included in the S1 File. Lastly, we conducted a thematic analysis by describing relationships between codes and grouping them into overall themes [18]. We triangulated data from observations and the semi-structured interviews.

## 3. Results

### 3.1 Participant characteristics

We conducted 17 interviews from June 2021-April 2022, each lasting 23 to 38 minutes. Thematic saturation was reached after 13 interviews. Participant characteristics are shown in Table 1. Most participants had both clinical and administrative roles (n = 10, 59%). About half of the participants were women (n = 8, 47%), and the median years of experience was 18 (Range: 3, 40).

**Table 1:** Respondent characteristics.

Characteristics	n (%)
Gender, women	8 (47%)
Educational attainment	
Associate Degree	1 (6%)
Bachelor’s Degree	5 (29%)
Graduate or Professional Degree	11 (65%)
Department	
Peri-Operative Services	7 (41%)
Corporate Supply Chain	4 (24%)
Materials Management	2 (12%)
Contracts	1 (6%)
Central Sterile	1 (6%)
MDR Industry	2 (12%)
Roles	
Administrative only	7 (41%)
Administrative and clinical	10 (59%)

**Abbreviations**: MDR, medical device reprocessing.

### 3.2 Overview of MDR

**Fig 1:**
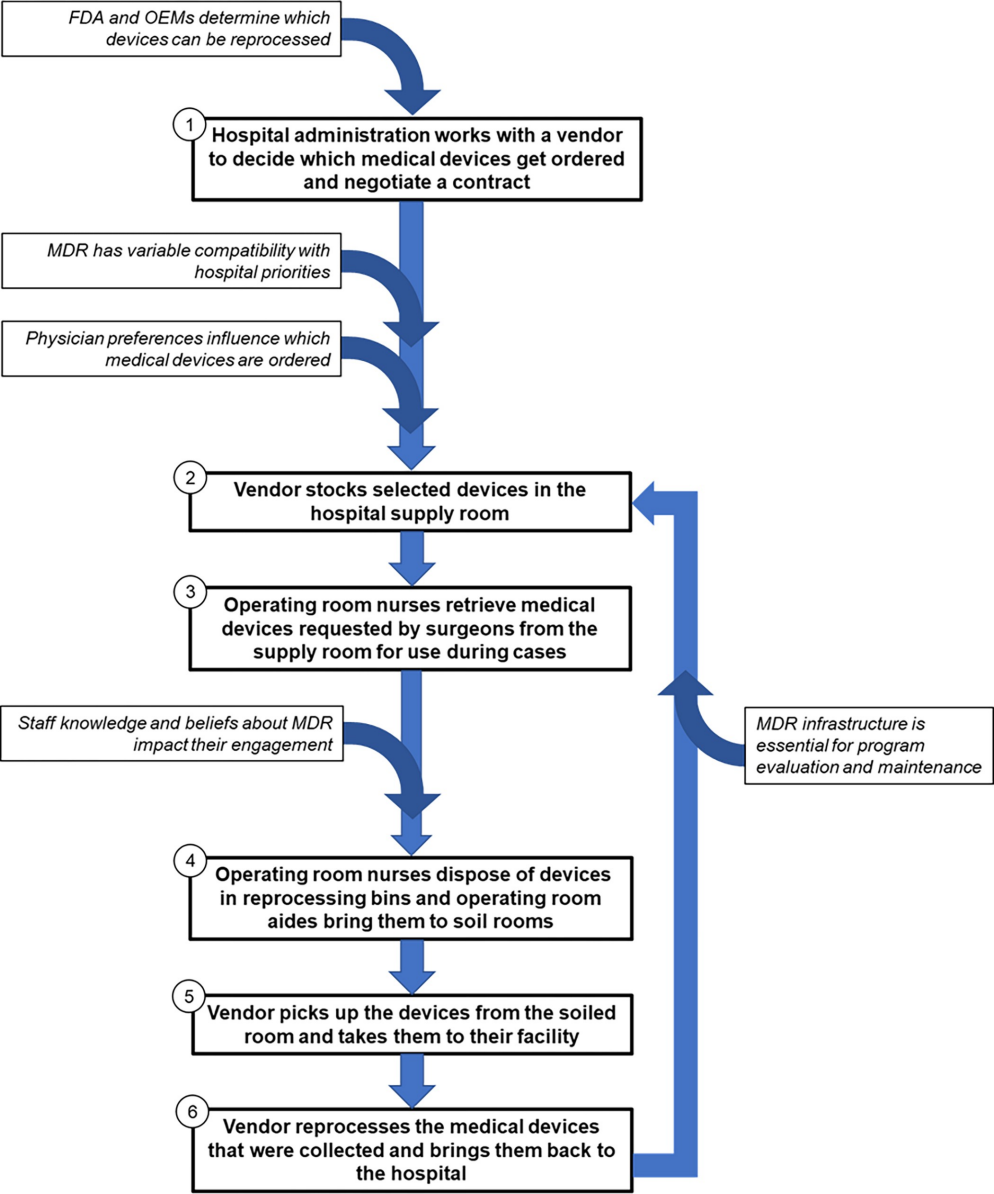
Conceptual model describing the six steps of implementing MDR and related themes. **Abbreviations**: MDR, medical device reprocessing; OEMs, original equipment manufacturers; FDA, Food and Drug Administration.

We identified six steps for implementing MDR in the health system (Fig 1). First, stakeholders at Corporate Supply Chain, Materials Management, and Contracts work with third-party MDR vendors to identify items of interest and to set up a contract. The contract may involve health systems selling used medical devices to third-party reprocessors, buying back reprocessed medical devices at a discounted rate, or both. If the contract involves buying back reprocessed medical devices, the vendor brings reprocessed devices to the hospital and stocks the selected devices in supply rooms. Nurses retrieve devices from the supply room for use during cases. After cases, nurses dispose of devices in designated MDR bins in the operating rooms, and, when full, operating room aides bring these bins to soiled utility rooms. The vendor picks up the used devices from soiled rooms and brings them back to their facilities to go through FDA-approved MDR procedures.

We identified five overarching themes that impact these six steps of MDR (Fig 1; Table 2). Respondents described that the FDA and OEMs determine which devices can be reprocessed, while hospital priorities and physician preferences influence which reprocessed devices get ordered. Once the contract is set up, variable staff knowledge and beliefs about MDR influences their motivations to participate in the program. Lastly, respondents emphasized that there was a lack of infrastructure for evaluating and maintaining MDR at Yale New Haven Health System.

**Table 2:** Barriers, facilitators, and recommendations to scale up MDR.

CFIR domains	Barriers	Facilitators	Recommendations
*FDA and OEMs determine which devices can be reprocessed*			
• Outer setting • Intervention characteristics	• Stringent FDA regulations • Forced obsolescence by OEMs	• FDA approval legitimizes MDR • Federal government’s interest in Right to Repair	• Implement changes in regulation processes, e.g., reducing the application burden to expand the pool of reprocessable devices • Implement federal policies that promote a circular economy in health care, e.g., require medical device design for reprocessability such as through modular components and durable materials that are easy to decontaminate and repair
*MDR has variable compatibility with hospital priorities*			
• Inner setting • Intervention characteristics	• Perception of decreased functionality • Lack of data on GHG emissions savings/costs	• Cost savings • Resilient supply chain • Culture of sustainability	• Bolster data availability from third-party vendors, such as life cycle assessments of GHG emissions and cost-effectiveness
*Physician preferences influence which medical devices are ordered*			
• Inner setting • Individuals • Intervention characteristics	• Physicians’ preferences for new single-use devices • Logistics of changing physician preference cards	• Physicians’ priority of cost and/or environmental savings	• Invite physicians to visit/provide videos of the MDR facility to learn about safety processes for ensuring sterility and functionality of devices • Provide physicians with data on functionality, sterility, environmental benefits, and cost savings
*Staff knowledge and beliefs about MDR impact their engagement*			
• Individuals • Intervention characteristics • Process	• Lack of knowledge on purpose, benefits and procedures of MDR • Low collection rates	• Increasing importance of environmental sustainability among staff	• Provide health system-wide training on MDR procedures • Post clear signs on MDR bins • Provide routine audit and feedback reports
*MDR infrastructure is essential for program evaluation and maintenance*			
• Inner setting • Process	• Lack of MDR program manager • Lack of data on GHG emissions to monitor environmental impact	• Vendor provides reports on pounds of averted waste and cost savings of MDR program	• Hire a full-time program manager for MDR • Create an auditing system for counting collection and buy-back rates to verify number of times an item has been reprocessed

**Abbreviations**: CFIR, Consolidated Framework for Implementation Research; MDR, medical device reprocessing; OEMs, original equipment manufacturers; FDA, Food and Drug Administration; GHG, greenhouse gas

### 3.3 FDA and OEMs determine which devices can be reprocessed

Respondents described that the FDA is responsible for approving single-use disposable devices and respective procedures for reprocessing. Stakeholders described that having FDA approval has legitimized the MDR industry and set high standards for ensuring patient safety, but has also created barriers for scaling up MDR nationally. One respondent from the Association of Medical Device Reprocessors described:

“*Having FDA seal of approval*, *it’s two sides of a coin*. *It’s legitimizing*, *but it’s also a barrier…we’re not just held to all the same standards as an OEM [new device]*, *we’re held to a higher standard…there are a lot of tests that OEMs [new devices] don’t have to do*, *mainly cleaning validation*, *sterilization validation and functional testing validation where our standards exceed what the OEMs’ [standards] are*.*”* (ID-17)

This respondent also described that conducting validation studies for cleaning, sterilization, and functional testing creates a significant financial barrier for expanding the pool of reprocessable medical devices.

In addition to the FDA, respondents described that OEMs play a pivotal role in MDR. Respondents described that OEMs are incentivized to make single-use devices that are not able to be reprocessed to maximize consumption and therefore profits. A stakeholder from Corporate Supply Chain explained:

“*If you are [a vendor] and you’re selling the LigaSure (laparoscopic electrocautery device)*, *you don’t want it to be used more than once or if it’s used on more than one patient*, *then that’s one additional one you haven’t sold…So the new LigaSures (laparoscopic electrocautery instruments) have a coating on the jaws that’s like a nonstick coating to keep the tissue from sticking to it*. *And if you reprocess it*, *that coating doesn’t survive*. *And so that device is not able to be reprocessed for that reason*. *And so that stuff is intentional by industry to foil reprocessing*.*”* (ID-07)

This stakeholder described that the OEMs purposefully design their devices so that reprocessing is not possible, which is what other respondents referred to as “forced obsolescence.” Respondents provided other examples of forced obsolescence by OEMs, such as software upgrades that would require reprocessors to seek additional approvals from the FDA.

Another way that OEMs inhibit MDR is by including language in their contracts that prohibit health systems from reprocessing their devices, even if the device is FDA-approved for MDR. One respondent from a reprocessing vendor noted:

“*OEMs sometimes work into their contracts with hospitals that they can’t purchase reprocessed devices or else they won’t sell them the device*. *That’s a real limitation to even getting reprocessed devices into the facility*.*”* (ID-16)

Despite these barriers, one respondent from the Association of Medical Device Reprocessors described that the federal administration’s interests may be aligned with MDR, such as through their support of the Right to Repair movement, which prohibits manufacturers from banning the repair of equipment and devices by individuals and independent repair shops in order to decrease electronic waste and consumer spending. They described:

“*I am pleased to see that the Biden administration and the FTC [Federal Trade Commission] issued a report on Right to Repair*, *and it was fairly critical of larger industries… And so shining a light on that [Right to Repair] is another example where I think we could increase reprocessing and the benefits that it brings*.*”* (ID-17)

As this respondent described, the Right to Repair movement has similar goals as MDR, which may work to promote federal policy that prohibits OEMs from barring MDR through forced obsolescence and anti-competitive contracts.

### 3.4 MDR has variable compatibility with hospital priorities

Respondents described how certain aspects of MDR were compatible with the health system’s priorities. For example, many respondents described that cost savings is an important priority for the hospital. One nurse manager described:

“*So they [hospital administration] would say if it’s more financially feasible to reprocess something versus buy something that’s [a new single-use] disposable*, *they would reprocess*. *They would take whatever is more cost effective*.*”* (ID-12)

Respondents described that another important priority for this health system is supply chain resiliency. This priority became particularly relevant during the SARS-CoV-2 pandemic, during which shortages in critical medical supplies negatively impacted health system staff and patient care. One stakeholder from Corporate Supply Chain described:

“*The other really important lesson from COVID was the fragility of the disposable supply chain*. *So if you buy a [reprocessable] device*, *like a pair of scissors for the operating room that you can sharpen and use for 30 years*, *if there’s a sudden problem with the disposable scissor market*, *you’re okay because you’ve got all the scissors you need…And so you can have a lower cost*, *more environmentally friendly*, *more resilient supply chain if you use reprocessable devices and reprocess the [single-use] disposable devices*.*”* (ID-07)

Respondents also reported that their hospital values sustainability initiatives, which is compatible with MDR. Participants described other programs in the hospital that focused on recycling and mitigating excessive waste. For example, one administrator at Corporate Supply Chain described:

“*For gynecology exams*, *there was a plastic [single-use disposable] speculum and had a light source that was battery operated…And then one of our guys was like*, *‘Are you crazy*? *We’re going to throw batteries out every time we do this*.*’ And we went alright we’re just going to plug stuff in with a separate battery source that’s rechargeable*. *So those are real life examples of how we kind of make our decisions*. *We like to use engagement from our staff*. *We like it to be everybody’s problem*.*”* (ID-03)

Despite these aspects of MDR that are compatible with hospital priorities, respondents described a few ways in which MDR was incompatible with their health system. For example, respondents described that a key barrier of MDR is variability in the functionality of devices after they are reprocessed, which creates skepticism that MDR has net environmental benefits if reprocessed devices are not usable. In order to calculate the environmental costs and benefits of reusing and/or reprocessing devices, one administrator and physician in Corporate Supply Chain described the importance of assessing the carbon footprint of cleaning and transporting these devices:

“*We’re driving trucks all over the state and interstate to get these [reprocessable] products to us*. *I think it would be interesting to understand what is the carbon footprint of that [re-sterilizing devices]*. *How would we even go about calculating it*? *Now*, *I think those types of things could really energize the group*.*”* (ID-03)

As this respondent noted, having data on life cycle GHG emissions associated with reprocessing and reusing devices is critical so that health systems can make informed decisions about which devices have environmental benefits.

Respondents also suggested that investigating new opportunities in MDR is not the main priority for the health system, especially because the hospital is still dealing with product availability issues related to the SARS-CoV-2 pandemic. One value analysis nurse reported:

“*I think if we were a little farther away from COVID*, *I think we could make more conscious decisions*. *But we are in such a knee-jerk reactive mode right now just to get product in the door so that clinical staff can do their jobs…Hopefully in a year or two from now supply chain will be unclogged and we’ll be able to make a little bit more conscious decisions around the environment when we make the purchasing decisions*.*”* (ID-09)

As this respondent described, the SARS-CoV-2 pandemic has resulted in surges in demand for equipment as well as interruptions in the supply chain, which has led the health system to secure supplies by any available means. Investigating ways for the hospital to institutionalize MDR may promote supply chain resiliency and buffer the impacts of future health system shocks, as noted by respondent ID-07.

### 3.5 Physician preferences influence which devices are ordered

Respondents described that physician preference for certain medical devices plays an important role in deciding which medical devices are ordered. One MDR vendor described:

“*They [physicians] might have a preference with a certain device that they’ve been trained on and have been using for a certain amount of years that they feel comfortable using*. *And even though we are supplying the exact same device [class]*, *there’s just this perception that it won’t function as correctly or it might be dirty…Then on the supply chain side*, *they’re saying*, *‘I know my doctors won’t use this*, *so I can’t move forward with it*.*’”* (ID-16)

Participants described that physicians would need access to key information to engage them in MDR. For example, a leader in Corporate Supply Chain and physician described:

“*If we can show significant cost savings*, *many physicians or surgeons do make decisions based on price*, *so cost data would be helpful*. *Data around efficacy*, *that the device is*, *quote unquote*, *just as good as a new device*, *that would go a long way to encourage physicians to do this [engage in MDR]*.*”* (ID-02)

Apart from gaining buy-in from physicians, respondents described that the process of changing physician preference cards in the system is complex logistically. One Corporate Supply Chain leader and physician described:

“*So*, *for instance*, *there’s a device called a LigaSure (laparoscopic electrocautery instrument)*. *In most hospital systems*, *that device has one kind of identifying number associated with it*, *a reprocessed version of that device has a different number*, *and when preference cards are built for the operating room*, *a surgeon would say*, *‘I have a preference*. *I like to use the 10-millimeter impact LigaSure (laparoscopic electrocautery instrument)*.*’ That preference card will get built using the initial device number*. *But then there’s not an effort to say*, *‘Hey*, *listen*, *would you be willing to use a reprocessed device*?*’ Which would require you to switch the person’s preference [card] to a different number*.*”* (ID-07)

Thus, in order to scale-up MDR in the hospital, respondents described that it will be important to present evidence on safety, quality, costs, life cycle emissions, and functional assurances to physicians, as well as systematically update and standardize physician preference cards in the system.

### 3.6 Staff knowledge and beliefs about MDR impact their engagement

Respondents noted that hospital staff need additional education on the purposes and procedures of MDR in order to engage them in the program. One nurse manager noted:

“*What was helpful for me was at one hospital I worked at*, *they were thinking about doing the [MDR] program*, *so they had the sales reps come in and give a very thorough video of how the stuff gets broken down [disassembled and cleaned]*, *how stringent it is*, *it’s an FDA process*. *Because I think a lot of people don’t realize what it has to go through to get it reprocessed*, *it’s not just some little thing being done in the backside of the garage*, *it’s an actual big deal*…*once I saw those videos and understood what they have to go through and what they have to throw out if it’s not working properly*, *it made a lot more sense to me*.*”* (ID-13)

By learning about MDR procedures and regulations, this respondent described that he gained confidence in the efficacy of the program, which motivated his engagement in MDR.

Many respondents noted that staff oftentimes do not know where to dispose of reprocessed medical devices in operating rooms, especially because there are numerous waste bins that each collect different items, e.g., sharps, medications, sharps with medications, fluids, recycling, general garbage, and medical devices for MDR. One nurse manager in peri-operative services described:

“*Each room is set up with a [reprocessing] bin*. *We were told as staff members what to put in and what not to put in*. *It was initially supposed to be a yearly refresher*, *but it has kind of fallen off*. *I still get questions every once in awhile asking about what goes in those bins*.*”* (ID-15)

As this nurse manager described, the refresher trainings on proper disposal of devices have “fallen off,” which negatively impacts collection rates of reprocessable medical devices.

Low compliance and collection rates ultimately create issues for ordering more reprocessed medical devices in the future. A respondent in Materials Management and Corporate Supply Chain described:

“*For example*, *we buy reprocessed tourniquet cuffs over here and I can tell you that buying reprocessed tourniquet cuffs has been a difficult process because the clinical team is not putting enough tourniquet cuffs into the reprocessing bins*, *which means that not enough tourniquet cuffs are getting back to the reprocessing plant*. *And therefore*, *when I place orders for more tourniquet cuffs*, *they’re not available*. *And then the order back-orders*. *And then that forces me to have a manual intervention where I need to force the system to buy new product instead of reprocessed product*.*”* (ID-04)

Depending on the contract, when collection rates are low, the MDR vendor will not send back as many reprocessed medical devices as needed, which forces stakeholders in Materials Management to buy additional new devices.

Thus, many respondents emphasized the importance of providing staff with annual training and information on where to throw out each medical device. One nurse manager described:

“*At [a different] facility where I worked*, *because there were multiple items and devices that were being reprocessed*, *they had posters everywhere to educate and show the staff where to dispose of the items and so they could be reprocessed*. *So all that information is very helpful so that it gets reprocessed correctly*.*”* (ID-14)

As these respondents described, providing education to staff on the purposes and procedures of MDR and posting clear signs describing where to dispose of devices are critical to improve compliance and collection rates.

### 3.7 MDR infrastructure is essential for program evaluation and maintenance

Respondents described a lack of infrastructure for evaluating and maintaining MDR programs. This presently requires staff who have a passion for sustainable health care to volunteer their time to spearhead these initiatives. Thus, participants described that having a project manager dedicated to MDR would be important to better maintain the program:

“*The problem that we have is*, *as an OR [operating room] manager*, *we have many different priorities*, *many different projects*, *many different issues that have to be addressed on a daily basis*. *And unfortunately*, *reprocessed products do not come even close to the top of the list*. *But having a dedicated project manager to help us convert more products from new ones to reprocessed*, *I think would be a big change*, *and it could give us some good results*.*”* (ID-04)

Respondents described that having a formal project manager could also help the hospital monitor the program so that improvements can be made. For example, respondents from Corporate Supply Chain and Materials Management described a need to be able to track reprocessed medical devices that have been used, collected, and reprocessed:

“*From a supply chain perspective*, *I would say that a concern would be whether or not they [MDR vendor] are actually adequately counting the number of times that it is being reprocessed*, *whether we are effectively collecting the items to reprocess*, *whether the company is actually fulfilling their obligation to pick it up*, *how we are maintaining the program*.*”* (ID-06)

Other respondents also described that providing progress reports to staff can facilitate their continued participation in the program. One administrator described:

“*So if you share with teams*, *this is how much we saved and how many pieces of equipment we diverted from the landfill and be very proactive with that information*, *people get engaged in that like*, *‘Wow*, *we diverted X number of pounds*, *that’s awesome*, *let’s see if we can divert more*.*’”* (ID-01)

Notably, the respondent from the MDR vendor described key progress metrics that they report back to hospitals, such as pounds of waste diverted from the landfill, how many trees were associated with collection efforts of the hospital, and carbon footprint diverted through carbon neutral shipping. Although these metrics may be available to the health system, other respondents described that they would need to be bolstered with life cycle GHG emissions data to provide further quantitative evidence on environmental impact.

## 4. Discussion

MDR has the potential to decrease de novo device manufacturing, hospital procurement and solid waste disposal costs and pollution; however, only a small proportion of devices that are approved for reprocessing are actually reprocessed [6, 7]. Thus, we conducted a qualitative study to explore the acceptability and feasibility of scaling up MDR in an academic medical center in New England. We identified barriers and facilitators of MDR related to the FDA, OEMs, hospital priorities, physician preferences, staff knowledge and beliefs, and hospital infrastructure. Based on our findings, we have outlined several recommendations that target these factors so that the environmental and financial benefits of MDR can be realized (Table 2).

First, our findings suggested that state and federal policy change is warranted to scale up MDR across health systems. Stakeholders described how having the “FDA seal of approval” has benefited the MDR industry by enforcing high standards to ensure patient safety. However, respondents described how OEMs use tactics to prevent health systems from engaging in MDR so that they can maximize profits, such as by designing medical devices to be obsolete after one use and including language in contracts that prohibits them from buying reprocessed devices. In this way, OEMs aim to uphold the “linear economy” paradigm, through which materials are extracted from the environment to make a device that is intentionally designed for one use [19]. On the other hand, MDR aims to shift from a “linear economy” to a “circular economy,” whereby devices are not discarded after one use, but are instead cleaned and remanufactured to preserve durability for reuse, repurposed when reuse is no longer possible, and recycled as a last resort [7]. Thus, creating federal policy that promotes a circular economy in the health care industry is critical [20]. Drawing on the Federal Trade Commission’s recommendations, the Right to Repair movement may be beneficial to the MDR industry, as both aim to prohibit OEMs from banning individuals and third-party companies from repairing devices to decrease de novo device manufacturing, consumer spending, and solid waste pollution [7, 21]. Future studies that critically analyze current policies related to MDR are warranted to develop a framework for expanding MDR in the US.

Our findings also suggested that interventions targeting the health system are warranted to expand MDR. For example, respondents described that health systems need access to better assessments of MDR to motivate their engagement. Life cycle assessments offer a strategy for evaluating which devices are good candidates for MDR. Life cycle assessments capture inputs of materials and emissions throughout a device’s life cycle (including extraction of raw materials, manufacturing, transportation, use/reuse, and waste disposal management), and quantify GHG emissions in units of carbon dioxide equivalents [7, 12, 22, 23]. Thus, vendors providing health systems with life cycle impact assessments of various medical device alternatives approved for MDR could aid program adoption [24]. However, more robust, transparent, standardized data on GHG emissions associated with single-use disposable, reprocessed single-use, and reusable medical devices are needed to facilitate decision-making [7].

We also identified interventions targeting individual practitioners to scale up MDR. Respondents described that a key barrier of MDR is physician preference for single-use devices, and providing data on efficacy, functionality, environmental benefits, and cost savings is critical to gain their buy-in. Many respondents described that they were skeptical about the safety of reprocessed devices; however, the US Government Accountability Office 2008 congressional report (GA-08-147) on the safety of reprocessed medical devices found that “FDA oversight has increased, and available information does not indicate that use presents an elevated health risk” [25]. Similarly, respondents described that nursing staff require additional information on the purpose and procedures of MDR to facilitate proper disposal of devices and collection rates. Stakeholders’ perspectives of the validity of evidence supporting a particular intervention is a key determinant of successful implementation [10]. Thus, providing ongoing system-wide education programs is paramount to successful scale-up of MDR.

Stakeholders noted that creating and disseminating progress reports to hospital staff is vital to facilitate sustained engagement. At this institution, vendor reports of total cost savings and solid waste diverted are shared with procurement and operational leadership, but are not routinely disseminated to staff. Respondents suggested that these reports should be distributed to staff and include metrics related to program performance (e.g., collection rates) and achievements (e.g., GHG emissions mitigated, pounds of waste diverted from the landfill, and cost savings). In a systematic review of audit and feedback reports in health care settings, the authors concluded that reports were most effective when they were provided routinely, feedback was give both verbally and in writing, and there were clear targets and an action plan for improvement [26]. Thus, health systems implementing MDR would benefit from providing audit and feedback reports to stakeholders on a regular basis through in-person team meetings and written emails. These reports should include metrics of performance alongside targets, such as by presenting collection rates alongside the target collection rate of 100%. In so doing, teams can create action plans for improving their performance.

Respondents described needing a dedicated program manager for MDR. Stakeholders explained that the onus of sustainability projects falls on individuals who are passionate about pollution prevention and willing to volunteer their time, which could result in burnout and suboptimal program efficacy. Previous studies have also identified that having a dedicated program manager who works with multiple stakeholders is critical to effectively implement programs in health systems [10, 27]. This program manager could facilitate continuing education on MDR and proper disposal, work with Corporate Supply Chain and Materials Management to prioritize sustainability in purchasing decisions, work with MDR vendors and Corporate Supply Chain to create and disseminate life cycle assessments, and create and disseminate audit and feedback reports to hospital staff.

Our study was strengthened by sampling respondents from various stakeholder groups, including industry stakeholders, hospital leaders, and clinical staff, until thematic saturation was achieved across groups. Our use of in-depth interviews and observations enabled us to triangulate our findings, improving the validity of our interpretations. Nonetheless, our study might be at risk for social desirability bias insofar that respondents spoke about MDR in an overly favorable way to align with our research objectives. However, participants did describe critiques of MDR, suggesting that social desirability bias, if present, was minimal. We did not interview any stakeholders from the FDA or OEMs; given the importance of these stakeholders in MDR, future studies should consider exploring their perspectives. This study was also based at a single health system in the US. Although this health system consists of a variety of settings, including a large academic medical center, small community hospitals, private hospitals, and ambulatory procedure centers, generalizability to other health systems may be limited. Further research is warranted, e.g., in Europe, where regulatory bodies, financial incentives, culture, and practices may differ. Lastly, this study focuses on reprocessing medical devices through third-party vendors, and additional studies focused on identifying barriers and facilitators of re-sterilizing reprocessable devices within the health system should also be conducted given its potential to decrease hospital waste.

## 5. Conclusion

This qualitative study explored stakeholders’ perspectives on MDR in an academic health system in New England. Our findings provide insights for MDR programs at health systems across the US. Implementing federal policies that promote a circular economy in the health system; assuring industry provision of accurate and transparent life cycle assessments of reprocessed medical devices; educating physicians and hospital staff on MDR processes, safety, cost-effectiveness, and environmental benefits; and providing stakeholders with routine audit and feedback reports are pivotal for scaling up MDR programs. Employing a dedicated program manager for MDR is vital to coordinate these efforts and maintain the program.

## Supporting information

S1 File Deductive and inductive codes, organized by consolidated framework for implementation research domain. (DOCX)
