# Supplementary material for: Stakeholder perspectives on scaling up medical device reprocessing: A qualitative study
Source: PLoS One. 2022 Dec 30;17(12):e0279808. doi: 10.1371/journal.pone.0279808 (PMC9803114; doi:10.1371/journal.pone.0279808)
Supplement: S1 File — (DOCX) [file pone.0279808.s001.docx]

Supplemental File 1. Deductive and inductive codes, organized by Consolidated Framework for Implementation Research domain.

| **Code** | **Description** |
| --- | --- |
| **Characteristics of individuals** | |
| Knowledge & Beliefs about climate change | Individuals’ attitudes toward and value placed on climate change as well as familiarity with facts, truths, and principles related to climate change, including hospital's contribution to climate change/pollution |
| Knowledge & Beliefs about MDR | Individuals’ attitudes toward and value placed on MDR as well as familiarity with facts, truths, and principles related to MDR. Includes advantages and disadvantages of MDR |
| Other personal attributes | A broad construct to include other personal traits such as tolerance of ambiguity, intellectual ability, motivation, values, competence, capacity, and learning style. For example, personal priority of environment conservation, burnout |
| **Inner setting** | |
| Hospital mission & priorities | Norms, values, and priorities of the hospital, eg., providing safe care to patients, cost-effectiveness, efficiency |
| Improvement Climate | The extent to which all staff work together to identify problems and create solutions. All staff feel responsible for quality improvement |
| Relative Priority of MDR | Individuals’ and/or teams' shared perception of the importance of MDR within the hospital. Includes discussions how certain OR teams prioritize recycling/reprocessing more than others. Includes discussions of how reprocessing PPE during COVID was prioritized because it was a time-sensitive crisis, but reprocessing other medical devices doesn't take that same priority |
| Sustainability culture | Extent to which sustainability is prioritized in the hospital, eg, other green initiatives |
| Access to Knowledge & Information about MDR | Ease of access to digestible information and knowledge about MDR and how to incorporate it into work tasks. |
| Available Resources | The level of resources dedicated for implementation and on-going operations, including money, training, education, physical space, and time. |
| Existing processes | Existing processes for ordering medical devices, including reprocessed medical devices |
| Leadership Engagement | Commitment, involvement, and accountability of leaders and managers with MDR and/or other green initiative (recycling programs, etc.). Also includes mentions of physician preferences for certain medical devices |
| PPE reprocessing project | Mentions of the program that reprocessed PPE during the pandemic--code the whole conversation in 1 big chunk |
| **Intervention characteristics** | |
| Cost | Costs of MDR and costs associated with implementing MDR including investment, supply, and opportunity costs. For responses that mention how MDR might be beneficial by saving money, code as "knowledge and beliefs about MDR" instead |
| Design quality & packaging | Perceived excellence in how reprocessed medical devices are bundled, presented, and assembled. Also includes mentions about how reprocessed medical devices are higher or lesser quality compared with single use disposables |
| Evidence strength & quality | Stakeholders’ perceptions of the quality and validity of evidence supporting the belief that MDR will have desired outcomes. Includes mentions of needing more evidence to know the cost benefits and environmental benefits of MDR |
| Trialability | The ability to test MDR on a small scale in the organization, and to be able to reverse course (undo implementation) if warranted. |
| **Outer setting** | |
| Cosmopolitanism | The degree to which an organization is networked with other external organizations. E.g., Northeast Purchasing Coalition (NPC) |
| External Policy & Incentives | A broad construct that includes policy and regulations (governmental or other central entity), external mandates, recommendations and guidelines, pay-for-performance, collaboratives, and public or benchmark reporting. E.g., FDA, regulatory bodies that determine which devices are safe for reprocessing |
| Medical device supply chain | Any mention of the medical device supply chain during the pandemic or during other times, suggesting that the medical device supply chain may be unpredictable/fluctuate |
| Medical or nursing associations | Mentions of medical associations or nursing associations that prioritize sustainability |
| **Process** | |
| Engaging | Thoughts on how to attract and involve appropriate individuals in the implementation and use of MDR through a combined strategy of social marketing, education, role modeling, training, communication, and other similar activities. |
| Executing | Thoughts on how to carry out the implementation of MDR according to plan, eg through constant reminders, clear labeling of trash bins, continued motivation of staff |
| Formally Appointed Internal Implementation Leaders | Extent to which it could be effective to appoint individuals from within the hospital to be responsible for implementing MDR (eg, coordinator, project manager, team leader, or other similar role). |
| Planning | Thoughts on how to plan for implementing MDR in advance, such as setting up contracts between YNHHS and Stryker or other groups, changing hospital policies, changing current infrastructure. |
| Reflecting & Evaluating | Thoughts on quantitative and qualitative feedback about the progress and quality of implementing MDR accompanied with regular personal and team debriefing about progress and experience. |
